# Costs of Dengue to the Health System and Individuals in Colombia from 2010 to 2012

**DOI:** 10.4269/ajtmh.14-0386

**Published:** 2015-04-01

**Authors:** Raul Castro Rodriguez, Katia Galera-Gelvez, Juan Guillermo López Yescas, Jorge A. Rueda-Gallardo

**Affiliations:** Department of Economics, Universidad de los Andes, Bogotá, Colombia; Sanofi Pasteur Latin America, Mexico DF, Mexico

## Abstract

Dengue fever (DF) is an important health issue in Colombia, but detailed information on economic costs to the healthcare system is lacking. Using information from official databases (2010–2012) and a face-to-face survey of 1,483 households with DF and dengue hemorrhagic fever (DHF) patients, we estimated the average cost per case. In 2010, the mean direct medical costs to the healthcare system per case of ambulatory DF, hospitalized DF, and DHF (in Colombian pesos converted to US dollars using the average exchange rate for 2012) were $52.8, $235.8, and $1,512.2, respectively. The mean direct non-medical costs to patients were greater ($29.7, $46.7, and $62.6, respectively) than the mean household direct medical costs ($13.3, $34.8, and $57.3, respectively). The average direct medical cost to the healthcare system of a case of ambulatory DF in 2010 was 57% of that in 2011. Our results highlight the high economic burden of the disease and could be useful for assigning limited health resources.

## Introduction

Dengue fever (DF) is an acute viral infection transmitted by female *Aedes aegypti* mosquitoes. In most cases, the disease follows a mild course with flu-like symptoms, severe headache, and aching joints and muscles.^1^ However, a small proportion of patients goes on to develop the potentially fatal hemorrhagic form of the disease.^2^

The disease is found in tropical and subtropical regions of the world.^3^,^4^ It is currently the most important arbovirus in the world in terms of morbidity, mortality, and economic burden.^5^ In South America, the disease became rare in the 1970s because of the eradication of the mosquito vector as a result of efforts to control yellow fever, another arbovirosis.^6^ However, when the eradication program ended, *Ae. aegypti* and DF spread once more, and the disease is now endemic in many Latin American countries.^7^ Indeed, the annual incidence of infection in the Americas has increased from 16.4 per 100,000 population at risk in the 1980s to 71.5 per 100,000 population between 2000 and 2007.^7^ In Colombia, for the entire period between 1978 and 2010, just over 1,020,000 cases were reported, corresponding to an average of 160.8 cases per 100,000 population per year,^8^ whereas in 2010, the last epidemic year, 606.2 cases per 100,000 population were reported (153,165 cases in total) according to the National Public Health Surveillance System (Sistema Nacional de Vigilancia en Salud Pública [SIVIGILA]^9^) database.

DF has, thus, become an important health issue in Colombia and represents a substantial economic burden on both the healthcare system and the individual patient. Despite the growing importance of DF, few studies have attempted to quantify the economic burden of the disease at a national level in Colombia. In a study based on a microcosting analysis of data from two Colombian cities (Monteria and Neiva), Castañeda-Orjuela and others^10^ estimated a direct medical cost of $79.17 for an ambulatory case, a cost of $733.32 for a case requiring hospitalization, and a cost of $1,160.56 for a case of dengue hemorrhagic fever (DHF; data for 2012). Shepard and others^11^ extrapolated more detailed data from other countries to infer a total economic cost of $185 per ambulatory case and $772 per hospitalized cases (data from 2010).

In view of the lack of direct data on a national level, this study was undertaken with the objective of quantifying the various components of the costs of a dengue case in Colombia. The method of data collection and analysis also enabled these costs to be tracked over a 3-year period encompassing an epidemic year (2010) and two non-epidemic years (2011 and 2012) to provide insight into how costs vary with disease incidence. Detailed information on the costs of dengue in Colombia would allow a more efficient allocation of resources for the control of dengue.

## Methods

The costs per case of DF in this study were derived from direct medical costs incurred by the Colombian healthcare system (including medical appointments, treatments, and other services), direct medical costs incurred by households (including copayment of medical expenses), direct non-medical costs incurred by households (such as travel expenses), and indirect costs incurred by households (for example, lost productivity).

The study combined information derived from national governmental agencies for each year of the study (2010–2012) to calculate direct medical costs to the state and information obtained through a survey of a population-based sample to estimate costs to households. This survey was conducted in 2012, and thus, costs per case derived from this survey refer to 2012.

### Colombian healthcare system.

Health coverage in Colombia is provided under the General System of Health and Social Security (Sistema General de Seguridad Social en Salud [SGSSS]).^12^ Under this mixed system, there is a contributive plan for those who work and are able to pay insurance premiums to private insurers and a subsidized plan for those who are unable to pay, with subsidies allocated according to a poverty scale. In both cases, medical assistance is subject to copayment or sliding-scale fees. Those who do not qualify for subsidies or are unable to pay the contributive plan are required to pay for their healthcare, although there is a Basic Health Plan (Plan de Atención Básica [PAB]) that covers all citizens for catastrophic illnesses, including DF, regardless of health coverage. In 2011, 96% of Colombians had some form of health coverage.^12^

### Governmental sources of information.

The direct medical costs to the healthcare system were derived from the Individual Registries for Health Service Provision (Registro Individual de Prestación de Servicios en Salud [RIPS]). The RIPS is a national database run by the Colombian Ministry of Health and Social Protection that captures data (including unit costs) on medical visits, medication and associated procedures, and other services for every patient in Colombia. Data from 2010–2012 pertaining to dengue-related diagnoses were extracted using the following International Classification of Disease codes (10th Edition): A90X (DF [classical dengue]), A91X (DHF), A928 (other specified mosquito-borne viral fevers), A929 (unspecified mosquito-borne viral fever), A930 (Oropouche virus disease), A931 (sandfly fever), A932 (Colorado tick fever), A94X (unspecified arthropod-borne viral fever), R500 (fever with chills), R501 (persistent fever), and R509 (unspecified fever). Entries not coded as DF or DHF were retrieved and reviewed for laboratory studies (blood workup and immunoglobulin M [IgM]) and related procedures (oral rehydration, paracetamol, and endovenous fluid administration in the case of hospitalized patients) according to an algorithm validated by a physician with expertise in the disease.

Deaths caused by dengue were identified from the SIVIGILA database for 2010–2012. This database was established in 2006 as a tool to guide public health policy and planning, and it includes only confirmed cases.^9^ It is mandatory for healthcare providers (including analysis laboratories) to notify the SIVIGILA of certain events, including.

For the purposes of calculating lost earnings because of premature death, a monthly income of $511.4 was calculated to be the average wage in the capital municipalities of 13 departments covered by the survey in 2012 according to the Gran Encuesta Integrada de Hogares (GEIH; Departamento Administrativo Nacional de Estadística [DANE]; http://www.dane.gov.co/files/investigaciones/fichas/Gran_encuesta_integrada_hogares.pdf), with a discount rate of 3% to account for future increases.

### Center of Studies on Economic Development 2012 population-based survey.

The data on expenses borne by the households were derived from a population-based survey undertaken by the Center of Studies on Economic Development (Centro de Estudios sobre Desarrollo Económico [CEDE]) in the last quarter of 2012. The survey used a written questionnaire with a broad range of questions that addressed many facets of DF, including knowledge, attitudes, and practices, in addition to economic aspects (a sample questionnaire is provided in Supplemental Material). The questionnaire development process included two pilot studies to test and adjust the questions. Questions relevant to the calculation of costs included employment status at the time of illness (average salary information was derived from the GEIH survey as described above), type of social security affiliation, date of admission to the hospital, and absenteeism from work or school because of illness. Questions were structured to obtain a breakdown of direct medical costs (expenditure on medicines, tests, and doctors' fees) and direct non-medical costs (transport, food, and lodging).

The study sample was drawn from a population of approximately 24 million inhabitants in municipalities below an altitude of 1,800 m (transmission is not thought to occur above this altitude^13^) according to a population projection based on the 2005 DANE census. Sample selection was a three-stage process. First, six departments (equivalent to US political states) corresponding to those with the highest prevalence of dengue cases were selected from the 13 departments that were surveyed according to the SIVIGILA data for 2011. Collectively, these departments accounted for 51.4% of the national cases of DF in 2012. Second, four municipalities were randomly chosen per department from those municipalities that represented 80% of dengue cases within that department. Third, households affected by DF or DHF were randomly selected from entries in the SIVIGILA database in the first half of 2012. The number of households selected per municipality was weighted to proportionally represent the total number of dengue cases within the department. After making initial telephone contact (where possible), interviewers visited each selected household and completed the questionnaire with the head of the household or another adult.

The sample size of the CEDE survey was calculated to achieve a precision in the difference between the true population value and sample value of 5% and a relative SE of 10%. The 95% confidence intervals (95% CIs) encompassing the real proportions of DF of the population were estimated for a proportion of 0.5. A correction of 1.5 for cluster design effect was applied. This yielded a sample size of 1,107 (for a total of 55,063 cases reported in SIVIGILA) for DF and 458 (for a total of 9,026 cases reported) for DHF.

### Calculation of costs.

All costs were calculated in pesos and then converted to US dollars (US$) using the average exchange rate for 2012 (1,798.23 pesos per 1 US$). (Exchange rate was according to Superintendencia Financiera de Colombia, 1.3.6 Serie histórica empalmada de datos. Promedio anual, 2013.) The direct medical cost of a case was the sum of the medical costs to the healthcare system and the household. The medical cost to the healthcare system for each year of the study was calculated by identifying diagnostic procedures, treatments, and other medical services used by patients selected from the RIPS database according to the Guideline for Integrated Care of the Dengue Patient^14^ and adding together the unit costs for those interventions. A sensitivity analysis was performed on these data using a bootstrap method.^15^ For each type of cost in each type of dengue, the original sample (used to calculate the average cost) was sampled to get 10,000 bootstrap resamples. This resulted in a vector size of 10,000, with the mean of every resample equal to the mean of the original sample assuming an empirical distribution. Then, the vector size of each type of cost was added to calculate the overall cost, and the 95% CI was estimated using the quintiles method. The direct medical cost to the household (for 2012) was determined using findings from the CEDE survey and consisted of the cost of hospitalization, physician fees, copayment or moderating quota (that is, a contribution to the cost from the insured person), laboratory tests, and treatments.

The direct non-medical cost to the household (for 2012) was calculated by adding transport costs, lodging and meals when accompanying the patient to other municipalities, caregivers, housing arrangements, funeral fees, and other household expenses. If a field was left blank, no expense was considered to have been incurred.

The indirect cost was the cost of lost productivity because of the patient's and/or caregiver's absenteeism from work calculated as the product of the daily earnings at the time of disease and the number of days of work lost. No cost was applied to days of school missed by sick children, although time taken off by adults to care for them was included.

Lost income because of death was calculated as the sum of income at the time of death and the projected duration of the patient's working life, assuming that they would have worked until the retirement age of 60 years old for men or 55 years old for women (after 2014, the new retirement age is 62 years old for men or 57 years old for women) adjusted for the probability of death from other causes. For deaths in childhood, it was assumed that the child would have worked from the age of 18 years old.

## Results

The numbers of ambulatory and hospitalized DF cases in the SIVIGILA system were 107,016 and 36,404, respectively, in the epidemic year of 2010; 19,366 and 11,970, respectively, in 2011; and 33,054 and 22,719, respectively, in 2012. Thus, 25% of all reported cases of DF in 2010 were hospitalized compared with approximately 40% of all cases in 2011 and 2012. There were 9,745 cases of DHF in 2010, 1,303 in 2011, and 1,465 in 2012.

### The 2012 CEDE survey.

Of 1,107 households with DF initially selected for interview, 1,089 responded to the interview and had at least one member of the household who had DF. For DHF, 394 respondents of 458 initially selected households provided data. Of the patients across all categories (ambulatory or hospitalized DF and DHF), 90–95% had either company health insurance or subsidized company health insurance. The mean ages of patients with DF and DHF were approximately 21 and 18 years old, respectively (Table 1). Of note, 52% of DF cases (ambulatory or requiring hospitalization) and 62% of DHF cases were children ages 14 years old or under. In the SIVIGILA database, 48% of 55,704 cases of DHF reported in 2012 were patients 14 years of age or under, whereas 55% of 1,462 cases of DHF were patients 14 years of age or under. According to the results of the 2012 CEDE survey, the number of productive days lost was almost 50% higher for hospitalized DF compared with ambulatory DF. A similar number of productive days was lost for hospitalized DF and DHF.

### Direct medical cost per case to the Colombian healthcare system between 2010 and 2012.

The average direct medical cost per case to the healthcare system of ambulatory DF was almost two times as high in 2011 than in the other 2 years (Table 2). The main driver for this difference was the greater cost of treatment. For hospitalized DF, a greater cost was also observed in 2012, although in this case, the main driver was the cost of other services (such as costs of hospital rooms, patient transfer, and use of medical equipment). The relative year-to-year variations were smaller in the case of DHF.

The results show a clear escalation in the cost of dengue according to disease severity. The average direct medical cost to the healthcare system of DHF was approximately 30, 20, and 40 times the cost of an ambulatory case in 2010, 2011, and 2012, respectively.

Table 2 also includes the 95% CIs estimated by a bootstrap analysis. The uncertainties in the estimates as indicated by broader CIs were observed for other services rather than treatment costs and for DHF compared with ambulatory and hospitalized DF.

### Costs incurred by households in 2012.

The direct medical costs, direct non-medical costs, and indirect costs all increased from ambulatory DF to hospitalized DF and finally, DHF (Table 3). In the case of direct medical costs, the principal driver in the differences observed was the cost of hospitalization. In the case of direct non-medical costs, the difference between hospitalized DF and DHF was small. Differences with regard to ambulatory DF were largely accounted for by transport, lodging, and food. It is also noteworthy that the contribution from indirect costs exceeded that from direct costs by more than a factor of 2 in the case of ambulatory DF and hospitalized DF and by more than a factor of 1.5 in the case of DHF.

### Total cost per case in 2012.

The total cost per dengue case in 2012 derived by summing the costs incurred by the healthcare system and the costs to the household (including indirect costs) was $202.3 for ambulatory patients, $497.9 for hospitalized patients, and $2,306.7 for patients with DHF. With increasing severity, the total cost is increasingly borne by the health system (86% in the case of DHF and 23% in the case of ambulatory patients with DF).

## Discussion

To our knowledge, this was the first study to rigorously calculate the cost of DF in Colombia, taking into account cost to both the healthcare system and patients. The study included comprehensive data from the centralized RIPS and SIVIGILA databases to derive costs to the healthcare system and data from a face-to-face survey of almost 1,500 households to derive costs borne by the individuals.

The most comprehensive study of the economic impact of DF in the Americas to date was performed by Shepard and others.^11^ Costs for all countries in the Americas were calculated by extrapolation from studies with appropriately reported cost data. The study that provided the most data included data for five countries in South America (Brazil, El Salvador, Guatemala, Panama, and Venezuela).^16^ The costs for these countries along with the extrapolated cost for Colombia and the comparable costs from this study are presented in Table 4. The methodology used in this study was similar to that used by Suaya and others.^16^ For ambulatory cases, the total cost per case derived from our study was more than $100 higher than the extrapolated value. A partial explanation for the difference between the extrapolated value and the value reported in our study for indirect costs could be in the number of productive days lost. In previous studies, patients attending school lost, on average, 4.2 days and patients working at the time of illness lost, on average, 6.6 days,^16^ whereas our ambulatory patients (both schoolchildren and workers) lost, on average, more than 8 days.

The direct medical costs incurred by households are lower than the medical costs to the healthcare system by a factor of between 3.7 and 4.7 according to setting. The fact that direct medical costs are incurred by households (particularly for treatments) is presumably a reflection of the copayment system in effect in Colombia. We note an almost two-fold higher contribution from indirect costs in this study compared with the extrapolation. The direct non-medical costs are almost threefold higher in this study compared with the extrapolation, although this still represents a relatively minor contribution to the total cost. The high indirect costs are consistent with the results of other studies with data on indirect costs^16^ and underline the importance of considering such costs when estimating the overall economic burden of disease.

The loss of income because of premature death seemed to increase from 2010 to 2012, presumably as a result of the lower average age of the patients who died. The reason for the decrease in the average age of death is not clear, particularly because the mortality rates for 2011 and 2012 were similar.

An interesting aspect of this study is that it captures changes in the direct medical costs per case over time. A number of studies have reported the economic burden of DF during epidemic periods,^17^,^18^ but to our knowledge, none have tracked the costs over a period of time. The lower average cost of treatment in 2010 is likely related to the large number of cases in this epidemic year. With the healthcare system being overburdened, finite resources have to be distributed among a larger number of patients. Thus, the time spent with each patient and the number of procedures are necessarily reduced. The difference is notable for both ambulatory and hospitalized cases but not so much for DHF, where the severity of the condition presumably means that the treatment protocols have to be followed in full, regardless of the burden on the system. In addition, epidemic years for DF may also coincide with epidemic years for malaria, which was the case in 2010,^19^ probably because the climatic conditions that favor the mosquito vectors for the two diseases are similar.^20^ The results for 2012, which was, like 2011, a non-epidemic year, showed low costs per case, like they were in 2010. For 2012, the number of cases used in the cost calculation (*N* = 134) was smaller compared with the number in 2011 (*N* = 3,932), although the number of overall cases was similar according to the SIVIGILA database. This discrepancy could be because the information required for the calculation had not been registered in the RIPS system at the time of data collection because of a lag time in data entry. If the cases that were entered in the system corresponded to the more severe ones, an artificial increase in the calculated cost would be expected to occur. However, given the low number of cases included in the analysis, the high cost may simply be a statistical artefact.

The study is subject to a number of limitations. The SIVIGILA database, like any other database, is subject to possible notification errors, ignored or incomplete fields, and lack of consistency in some variables. Moreover, the sensitivity and specificity of the reporting system may vary between epidemic years (resulting from overreporting because of other infections being mistaken for dengue together with increased media coverage) and non-epidemic years. Data collection is, nevertheless, a legal requirement. The sensitivity analysis for the direct medical costs to the health system showed the largest 95% CI and hence, the greatest potential for error in the calculation of the costs for other services, particularly in the case of DHF. In the case of the costs to households, the survey was conducted in 2012 in households that had had a case of DF in that year. These results were used to extrapolate to 2011 and 2010. It could be that the costs varied from year to year just as the direct medical cost to the healthcare system did. The survey sample was also based on patients who were recorded in the SIVIGILA system. Some individuals may have had mild DF and not sought medical care. Although such patients would not influence the average cost to the healthcare system per registered case, they would probably have lower direct non-medical costs and lower indirect costs. We also note that the survey sample was not fully representative for age groups or geographic regions, although the proportion of patients ages 14 years old or under with DF and DHF included in the survey was similar to the proportion reported in the SIVIGILA database. Finally, our study provided aggregate costs for all patients, although large differences have been reported in costs between adult patients and children in a study in Panama.^21^ However, the differences reported in that study could have resulted from the additional cost that was imputed for children who missed school (calculated using the cost of providing a day of public primary school). Our methodology did not include such a cost imputation.

In conclusion, the results of this study to directly assess the costs of DF in Colombia highlight the high economic burden of the disease and could be used as a guide for assignment of limited health resources.

## Supplementary Material

Supplemental Datas.

## Figures and Tables

**Table 1:** Characteristics of patients from the sample of households included in the CEDE survey

	Ambulatory DF (*N* = 462)	DF requiring hospitalization (*N* = 627)	DHF (*N* = 394)
Sex (male/female), %	52.6/47.4	50.6/49.4	50.6/49.4
Mean age, years	21.77	20.69	17.82
Occupation at the time when dengue was contracted			
Employed	124 (28.8%)	106 (16.9%)	57 (14.8%)
Studying	188 (40.7%)	262 (41.8%)	169 (43.9%)
Homemaking	39 (8.4%)	59 (9.4%)	31 (8.1%)
Unemployed	10 (2.1%)	9 (1.4%)	11 (2.9%)
Social security affiliation			
Company health insurance/prepaid	232 (50.2%)	221 (35.3%)	148 (38.5%)
Subsidized company health insurance	207 (44.8%)	371 (59.2%)	208 (54.2%)
Armed forces/police	13 (2.8%)	21 (3.4%)	19 (4.9%)
Other entity	8 (1.7%)	6 (1%)	5 (1.3%)
Productive days lost	8.32	13	14.41

Source: CEDE Survey (2012).

**Table 2:** Summary of average direct medical cost to the healthcare system per case over the study period

Year and type of cost	Ambulatory DF		Hospitalized DF		DHF	
	*N*	Average cost (US$; 95% CI)*	*N*	Average cost (US$; 95% CI)*	*N*	Average cost (US$; 95% CI)*
2010						
Total treatment costs†	78,667	52.4 (51.92–52.97)	4,252	164.6 (158.40–171.20)	2,712	216.0 (203.30–229.70)
Other services‡	264	0.4 (0.28–0.42)	416	71.2 (61.74–80.01)	6	1,296.2 (714.00–2,324.00)
Overall cost	78,667	52.8 (52.26–53.32)	4,252	235.8 (224.43–246.78)	2,712	1,512.2 (929.71–2,544.03)
2011						
Total treatment costs†	3,932	91.9 (89.61–94.30)	344	203.5 (167.20–251.60)	164	342.9 (259.00–435.50)
Other services‡	681	1.2 (0.94–1.33)	1,436	180.9 (159.70–174.80)	141	1,508.3 (1,263.00–1,705.00)
Overall cost	3,932	93.2 (90.74–95.46)	344	384.4 (333.94–418.67)	164	1,851.2 (1,586.00–2,071.80)
2012						
Total treatment costs†	134	46.2 (42.42–50.13)	35	89.6 (65.97–118.04)	19	303.7 (104.20–647.40)
Other services‡	903	1.3 (1.10–1.42)	1,476	137.6 (124.20–137.70)	91	1,680.7 (1,313.00–2,014.00)
Overall cost	134	47.5 (43.66–51.31)	35	227.1 (196.11–249.38)	19	1,984.4 (1,524.57–2,457.22)

Given that the samples of total treatment costs and other services for all types of dengue were independent and different sizes, there was no way to calculate the overall cost using the original samples. Therefore, the solution was to bootstrap each sample 10,000 times and create a vector size of 10,000, with the mean of every resample equal to the initial sample for all types of dengue. Then, the vector size of 10,000 of treatment cost and other costs was added depending on the type of dengue to calculate the total cost. With this new vector again of size 10,000, for each type of dengue, the CI was estimated using the quintiles method. Source: RIPS.

* Calculated using bootstrap analysis using the R statistical program.

† Includes medical appointments, treatments, and laboratory tests.

‡ Includes cost of hospital room, transfer to other care centers, and use of medical equipment.

**Table 3:** Summary of average costs (US$) incurred by households for 2012

Cost group and cost category	Ambulatory DF (*N* = 462)	Hospitalized DF* (*N* = 627)	DHF (*N* = 394)
Direct medical costs	13.3	34.8	57.3
Hospitalization	0	21.7	38.6
Physician fees	1.7	1.6	2.0
Copayment	1.6	0.5	0.7
Laboratory tests	1.8	1.3	3.1
Treatments	8.2	9.7	12.8
Direct non-medical costs	29.7	46.7	62.6
Transport			
Within municipality of residence	14.5	6.0	8.9
Outside municipality of residence	0	18.7	19.3
Nurses' and caregivers' fees	0.3	0.3	0.3
Lodging	0	3.8	14.2
Food	0	9.6	8.2
Child caregivers' fees	0.1	0.0	0.0
Changes to living quarters	0.1	0.0	0.0
Post-disease expenses	7.3	4.2	6.0
Other expenses	7.4	4.1	5.7
Indirect costs	111.8	189.3	202.4
Total	154.8	270.8	322.3

Averages calculated for all respondents. Source: CEDE Survey (2012).

* Excluding patients with a diagnosis of DHF.

**Table 4:** Comparison of the estimated average costs of dengue cases (US$) in this study with literature values

	Shepard and others*^11^					Shepard and others^11^: Colombia (extrapolated)†	This study: Colombia (direct study)‡
	Brazil	El Salvador	Guatemala	Panama	Venezuela		
Ambulatory cases							
Direct medical	49	27	24	78	118	66	67.1
Direct non-medical	18	2	10	25	18	11	29.7
Indirect	317	77	78	313	194	108	197.1
Total cost	383	107	111	416	331	185	293.9
Hospitalized cases§							
Direct medical	381	289	304	514	864	353	330.6
Direct non-medical	47	170	155	419	64	128	52.7
Indirect	460	99	72	404	310	291	310.5
Total cost	889	559	531	1,336	1,238	772	693.8

Source: RIPS and SIVIGILA databases, CEDE survey (2012), GEIH. Full description of sources is in the text.

* Corresponds to data collected by Suaya and others^16^ for 2004–2007, with most data collected in 2005 and adjusted to prices in 2010 using inflation statistics.

† Corresponds to extrapolated data for 2010.

‡ Corresponds to data for 2010.

§ Includes cases of DF that required hospitalization and DHF.
