# Supplementary material for: Costs of Dengue to the Health System and Individuals in Colombia from 2010 to 2012
Source: Am J Trop Med Hyg. 2015 Apr 1;92(4):709–14. doi: 10.4269/ajtmh.14-0386 (PMC4385762; doi:10.4269/ajtmh.14-0386)
Supplement: Supplementary file 1 [file SD2.pdf]

Universidad de los Andes	HOUSEHOLD SURVEY	CEIS Centro de estudios e investigación en salud FUNDACION SANTA FE DE BOGOTA

The information provided is strictly confidential and will only be used for statistical analysis

A. Control Data

1 **Municipality**

2 **Address**

3 **Telephone**

4 **Household** High trans. 1 Low trans. 2

B. Housing information

1 **Type of housing**

a. House	1
b. Room	2
c. Flat	3
d. Other type of housing (tent, shop, van, houseboat, natural refuge, bridge, etc)	4

2 **Main material used for outer walls**

a. Blocks, bricks, stone, polished wood	1
b. Earth, wattle and daub	2
c. Coated wattle and daub	3
d. Wattle and daub without coating	4
e. Rough wood, boards, cork	5
f. Prefabricated material	6
g. Guadua, bamboo, mat, other plants	7
h. Zinc, cloth, canvas, cardboard, tin, waste, plastic	8
i. No walls	9

3 **Main material used for floors**

a. Marble, wood, polished and varnished wood	1
b. Mat or wall-to-wall carpet	2
c. Tiles, vinyl, boards, brick.	3
d. Rough wood, boards, cork, other natural materials	4
e. Cement, gravel	5
f. Earth, sand	6

4 **Which of the following public, private or communal amenities does the housing have?**

a. Running water:	1
b. Sewer connection	1
c. Mains electricity	1
d. Piped natural gas	1
e. Propane gas (cylinders/tanks)	1
f. Landline phone	1
g. Refuse collection:	1

5 **Housing occupied by household is:**

Own property, but paying mortgage	1
Own property, inherited	2
Own property, fully paid	3
Rented or sublet	4
Usufruct	5
A squat	6

C. Living conditions of the household

1 **How many households are there in this housing?**

2 **How many people live in this household?**

	Total	Studying	Working
Less than 5 years old			
Between 5 and 11			
Between 12 and 18			
Between 19 and 50			
Over 50 years old			

3 **What type of sanitation does your household have?**

a. Toilet connected to sewer system	1
b. Toilet connected to septic tank	2
c. Toilet with no connection	3
d. Latrine	4
e. Tidal	5
f. There is no sanitation	6

4 **How do you dispose of household waste?**

a. There is a refuse collection service	1
b. Incineration	2
c. Land-fill	3
d. Thrown into the river, channel, ravine or lagoon	4
e. Thrown into the patio, mound, ditch or waste ground	5
f. Collected by an informal service (wheel barrow, cart, etc)	6

5 **The water for preparing food is obtained mainly from:**

Public or communal water supply	1
Well with pump	2
Well without pump, pool	3
Rain water	4
River, stream, spring	5
Public fountain	6
Tank truck/water carrier	7
Bottled water or water in other recipient	8

(2–8) Go to 7

6 **The water from the mains reaches your home:**

All the time	1	Sometimes	4
Some days	2	Never	5
Once a week	3	Other	6

7 **Do you store water in pans or recipients in your home?**

Yes, for more than 7 days	1	Don´t store	3
Yes, for less than 7 days	2		

8 **Do you store tyres, bottles and other recipients?**

Yes 1 No 2 → Go to 10

9 **How often do you clean these objects?**

Every week	1	Rarely	4
Every 15 days	2	Never	5
Once a month	3		

10 **Does the household have a sink, basin, or tank?**

Yes [1] No [2] → Go to 12

11 **How often do you wash it?**

Number of days [ ]

Never [0]

12 **How does this household prevent mosquito bites?**

Window nets [1]

Bed nets [1]

Fumigation [1]

Use of insect repellent [1]

Other, which? ________________ [1]

None [1] Go to D

13 **How much did the household spend last year on:**

Window nets $____________________

Repellents $____________________

Bed nets $____________________

Insecticide $____________________

D. Household information

1 **Age of head of household**

[ ] years old

2 **Sex of head of household**

1 [ ] Male 2 [ ] Female

3 **Marital status of head of household**

[1] Single

[2] Married/civil union

[3] Separated/divorced

[4] Widow(er)

4 **Level of studies of head of household**

[1] None [4] Higher education

[2] Primary [5] Technician

[3] Secondary [6] Technology

5 **Working status of head of household**

[1] Working → Occupation ____________________

[2] Homemaker ____________________

[3] Study

[4] Private means/retired

[5] Looking for work

[6] Permanent disability

[7] Other activity,

What? ______________

6 **Approximately how much did this household spend last month on:**

a. Food $____________________

b. Education $____________________

c. Health $____________________

d. Public services $____________________

e. Transport $____________________

f. Housing (rent, etc) $____________________

g. Leisure $____________________

h. Purchase of insecticides $____________________

i. Purchase of repellents $____________________

j. Others $____________________

7 **What is the monthly household income**

a. Head of household $______________

b. Income of other household members $______________

c. Rent $______________

d. Pensions $______________

e. Benefits $______________

f. Other income $______________

8 **Is the household affiliated to Sisben?**

Yes [1] → Level _______

No [2]

E. Practical Knowledge and Attitudes

1 **Have you heard about dengue fever?**

yes [1] No [2] → Go to 3

2 **Where have you heard of dengue fever? (wait for answer)**

Television [1] Health provider [1]

Radio [1] Friends/colleagues [1]

Papers [1] Neighbors [1]

Poster [1] In hospital [1]

Other, which? __________________ [1]

3 **Have received invitations to or participated in preventive or control activities for dengue fever?**

Yes [1] No [2]

4 **Do you consider dengue fever a problem?**

Yes [1] No [2]

5 **If you have heard about dengue fever, in your opinion, it is a serious, moderate or mild disease?**

Serious [1] Mild [4]

Moderate [2] Don´t know [5]

It can be all three [3]

6 **In your opinion: How is dengue fever transmitted between persons?** Interviewer: DONT READ RESPONSES

Mosquito? [1]

Direct contact [1]

Water [1]

Flies [1]

Dirty surroundings [1]

Rats, flees, pigs, other animals [1]

Others, specify________________ [1]

Don´t know [2]

7 **Can dengue fever be prevented?**

Yes [1] No [2]

Not sure [3]

8 **Generally, where do mosquitos lay their eggs and grow?** (Dont read responses)

Clean water [1] On wet clothing [1]

Dirty water [1] In plants [1]

Anything [1] Don´t know [1]

Other, specify____________________ [1]

9 **Have you seen larvae in your home at some time?**

Yes [1] No [2]

10 **What do you do at home to reduce the presence of mosquitos?** ***(Do not read the responses)***

Nothing	1	
Spray the house interior	1	
Clean refuse containers	1	
Cover the water tank	1	
Add chemicals to the water	1	
Keep animals in the water (fish, turtles)	1	
Use bacteria to combat larvae	1	
Use bed nets	1	
Kill mosquitoes, e.g. with chemicals or mosquito coil	1	
Personal protection with repellents	1	
Other, what? ______________________	1	

11 **What measures does the government take to control the mosquito that transmits dengue fever?**

Interviewer: *DO NOT read the responses*

Check water reservoirs	1		Dont know	1	
Add chemicals to the water	1		Nothing	2	
Spray the inside of the house	1				
Educate people	1				
Provide covers for recipients	1				
Provide fish/bacteria	1				
Fumigation	1				
Cut plants	1				
Collection of old tyres	1				
Other specify ____________	1				

12 **When was the last time you were visited by staff working on the eradication of the mosquito that transmits dengue fever?**

In the last month	1		More than a year ago	4	
2 and 6 months ago	2		Don´t remember/never	5	
6 and 12 months ago	3				

13 **Have you received support or materials (biological control) for elimination of dengue mosquitos or larvae? (bacteria, fish, etc).**

Yes	1		Specify ________________________
No	2		__________________________________

14 **What should the government do to improve control of dengue fever?**

Interviewer: DO NOT read the responses

a. Check the water deposits	1	
b. Add chemicals to the water	1	
c. Fumigate the inside of the house	1	
d. Educate people	1	
e. Provide covers for recipients	1	
f. Supply fish or bacteria to have in the water	1	
g. Fumigate the outside of the housing	1	
h. Cut down plants	1	
i. Other specify ___________	1	
j. Nothing	1	

F. Assessment scenarios

1 Dengue fever, transmitted by a mosquito, could negatively affect the quality of life of the members of the household and impact their health making them unable to perform normal activities and generating costs for the household (purchase of drugs, doctor visits, etc.)
If the national, regional, or municipal authorities undertook a project to completely eliminate this disease in this municipality, allowing the households to avoid high costs in prevention, disease treatment and loss of pay due to sick leave:
Would you be prepared to pay **$ 10** a month as a contribution to enable the authorities to eradicate this disease, by means of a services bill issued by municipality for five years?

Yes, I would pay for eradication of dengue fever [1] ➡ Go to 2 — No [2] ➡ Go to 4

2 **Are you sure of this answer, bearing in mind the importance of the health of your household members in their daily lives?**

Yes [1] ➡ Go to 3 — No [2] ➡ Go to 4

3 **And if the monthly sum to pay were $ 11.5, would you be prepared to pay?**

Yes [1] ➡ Go to 6 — No [2] ➡ Go to 6

4 **And if, instead of this sum, you had to pay $ 8.5, would you be prepared to pay?**

Yes [1] ➡ Go to 6 — No [2] ➡ Go to 5

5 **What is the main reason you would not be prepared to pay?**

Don´t think that I will become ill	1		Due to corruption	4	
Don´t believe in the authorities	2		No money available	5	
Others should have to pay	3		The monthly quota is very high	6	
Other reason	7		Which ? ______________________		

6 **Risk assessment**

A	1	
B	2	
C	3	

1	**Interviewer name**	2	**Name of supervisor**

G. Dengue fever morbidity (individuals who became ill in 2011)

(Use this format for individuals who became ill with dengue fever in 2011)

Name of interviewee ______________________________________Relation to patient __________________

1 **Name** ________________________________ Age __________ Sex Male 1 ☐ Female 2 ☐

Approximate date of disease ________________

2 **Identity document** ___________________________ Type cc ☐ TI ☐ Other ☐

3 **Relation to head of household**

1. Head ☐
2. Spouse ☐
3. Son/daughter ☐
4. Other relation ☐
5. Not related ☐

4 **Work situation on contracting the disease**

Working 1 ☐

Studying 2 ☐ → How many days of school were missed due to this disease? ☐

Homemaker 3 ☐

Looking for work: 4 ☐

Other activity 5 ☐ Which? ____________________________

5 **If working:** **NOTE: the monthly salary is the one received at the time of disease**

Monthly salary $__________________ Days off work due to the disease _______

6 **(NAME)…………….Were you affiliated to the social security system?**

EPS / PRE-PAID 1 ☐ Military / police 3 ☐ Affiliated 5 ☐

EPSS 2 ☐ Other entity 4 ☐ Not affiliated 6 ☐

7 **…………(NAME) Was hospitalized (a) (<u>considered hospitalized if in hospital for more than 8 hours</u>)**

Yes 1 ☐ →

No 2 ☐

Hospital name	Municipality	# Days

8 **…………(NAME) In total, how many days were you ill with this disease?**

Pre-sick leave complaints	**Off work**	Days with complaints after sick leave

9 **When …..(NAME) became ill with DENGUE FEVER, what were the costs to the household for:**

	In the POS as copayment	In the POS out-of pocket expenses	Own resources not insured	Self-medication
Hospitalization	$	$	$	
Copayment / Quota	$			
Physician fees / traditional physicians		$	$	
Transport costs within municipality		$	$	
Transport costs outside municipality		$	$	
Laboratory tests	$	$	$	
Nurses and carers fees		$	$	
Purchase of medicines	$	$	$	$
Lodging in other towns		$	$	
Food expenses in other towns		$	$	
Child carer fees		$	$	
Changes to living quarters		$	$	
Funeral expenses		$	$	
Post-disease expenses		$	$	
Other expenses	$	$	$	$

10 **Some of the members of the household of ………. stopped working or studying to care for the patient?**

Working 1 ☐ → How many days work missed? ______ What was their monthly salary? $_____________

Studying 2 ☐ → How many stopped studying? ___ In total, how many days did they miss? ___

No 3 ☐ **<u>Note</u>**: if more than one person stopped working, not the details on the back of the form

11 **Diagnosis for ………(NAME):**

Dengue fever 1 ☐ Dengue hemolytic 2 ☐ Died 3 ☐
